# Epidemiology of Schistosomiasis in the People’s Republic of China, 2004

**DOI:** 10.3201/eid1310.061423

**Published:** 2007-10

**Authors:** Xiao-Nong Zhou, Jia-Gang Guo, Xiao-Hua Wu, Qing-Wu Jiang, Jiang Zheng, Hui Dang, Xian-Hong Wang, Jing Xu, Hong-Qing Zhu, Guan-Ling Wu, Yue-Sheng Li, Xing-Jian Xu, Hong-Gen Chen, Tian-Ping Wang, Yin-Chang Zhu, Dong-Chuan Qiu, Xing-Qi Dong, Gen-Ming Zhao, Shao-Ji Zhang, Nai-Qing Zhao, Gang Xia, Li-Ying Wang, Shi-Qing Zhang, Dan-Dan Lin, Ming-Gang Chen, Yang Hao

**Affiliations:** * National Institute of Parasitic Diseases, Shanghai, People’s Republic of China; † Fudan University, Shanghai, People’s Republic of China; ‡ Nanjing Medical University, Nanjing, People’s Republic of China; § Hunan Institute of Parasitic Diseases, Yueyang, People’s Republic of China; ¶ Hubei Institute of Parasitic Diseases, Wuhan, People’s Republic of China; # Jiangxi Institute of Parasitic Diseases, Nanchang, People’s Republic of China; ** Anhui Institute of Parasitic Diseases, Wuhu, People’s Republic of China; †† Jiangsu Institute of Parasitic Diseases, Wuxi, People’s Republic of China; ‡‡ Sichuan Institute of Parasitic Diseases, Chengdu, People’s Republic of China; §§ Yunnan Institute of Endemic Diseases, Dali, People’s Republic of China; ¶¶ Ministry of Health, Beijing, People’s Republic of China

**Keywords:** Schistosomiasis, Schistosoma japonicum, cluster sampling survey, status, epidemiology, China, research

## Abstract

Results from the third nationwide cluster sampling survey on the epidemiology of schistosomiasis in the People’s Republic of China, conducted by the Ministry of Health in 2004, are presented. A stratified cluster random sampling technique was used, and 239 villages were selected in 7 provinces where *Schistosoma japonicum* remains endemic. A total of 250,987 residents 6–65 years of age were included in the survey. Estimated prevalence rates in the provinces of Hunan, Hubei, Jiangxi, Anhui, Yunnan, Sichuan, and Jiangsu were 4.2%, 3.8%, 3.1%, 2.2%, 1.7%, 0.9%, and 0.3%, respectively. The highest prevalence rates were in the lake and marshland region (3.8%) and the lowest rates were in the plain region with waterway networks (0.06%). Extrapolation to all residents in schistosome-endemic areas indicated 726,112 infections. This indicates a reduction of 16.1% compared with a nationwide survey conducted in 1995. However, human infection rates increased by 3.9% in settings where transmission is ongoing.

## Abstract 2

Although the number of human infections decreased, human prevalence increased from 4.9% in 1995 to 5.1% in 2004.

Schistosomiasis, which is caused by *Schistosoma japonicum*, is one of the most serious parasitic diseases in the People’s Republic of China despite a documented history >2,100 years. The first reported clinical case in modern China was made by an American physician in 1905 (*1*). On the basis of limited hospital-based data and fragmentary epidemiologic survey reports, schistosomiasis japonica in 1947 was endemic in 138 counties in China. The rural population at that time in those counties was ≈25.3 million, which was the at-risk population. The estimated number of people infected with *S. japonicum* was 5.3 million (*2*). Mao estimated that 32.8 million Chinese were infected with *S. japonicum* in the late 1940s (*2*). However, use of different sources and province-specific prevalence data showed higher estimates of the number of people infected and at-risk populations (*3**,**4*).

Since 1949, after the founding of the People’s Republic of China, large-scale epidemiologic surveys were conducted by Chinese health workers to identify schistosomiasis-endemic areas, prevalence and incidence of this disease, and number of deaths caused by *S. japonicum* infections. Results showed that schistosomiasis was endemic in 12 provinces, with an estimated 11.6 million people infected. There were 1.2 million infected cattle and an area of 14,300 km^2^ was infested by the intermediate host snail, *Oncomelania hupensis* (*5*).

Over the past 50 years, the ongoing national control program has made great progress in controlling this disease. To date, 5 of 12 formerly *S*. *japonicum*–endemic provinces and >60% of disease-endemic counties have reached the national criteria of transmission interruption, and the number of human infections has been reduced by >90% (*6*–*8*). However, in 2003, 110 counties had not yet reached the criteria for transmission control, i.e., the overall prevalence in disease-endemic villages of these counties was >1% (*9*,*10*). The epidemiology of *S*. *japonicum* in China and achievements made in its control have been reviewed (*11*). New data suggest that progress has stalled since the termination of the World Bank Loan Project (WBLP) for schistosomiasis control at the end of 2001 (*7*,*9*,*12*,*13*).

In 1989, the first nationwide schistosomiasis sampling survey was conducted by the Chinese Ministry of Health to determine the prevalence of *S. japonicum* in all regions of the country where transmission occurs (*14*–*16*). An estimated 1,638,103 people were infected (*8*,*16*). Six years later, the second nationwide schistosomiasis survey was completed. The number of people infected with *S. japonicum* had been reduced by >40% to 865,084 (*15*). Nevertheless, there is considerable concern that schistosomiasis has reemerged in some adjacent areas of hyperendemic regions in the new millennium (*9*,*17*–*19*).

The third nationwide cluster sample survey was conducted in 2004 to update epidemiologic data for schistosomiasis in China. The data generated can serve as benchmarks for design of a new framework of a national control program that includes mid-term and long-term goals and a more flexible strategy. The National Institute of Parasitic Diseases (IPD) at the Chinese Center for Disease Control and Prevention in Shanghai (*10*) was entrusted by the Ministry of Health to design, manage, and supervise this survey in close collaboration with specialized provincial institutions in the 7 provinces where *S*. *japonicum* was endemic.

## Materials and Methods

### Sampling Strategy and Study Population

The third nationwide schistosomiasis sampling survey covered all 7 schistosome-endemic provinces (Anhui, Hubei, Hunan, Jiangsu, Jiangxi, Sichuan, and Yunnan). The sampling unit was the administrative village (i.e., basic level of administration, often comprising >1 natural village), with only those villages selected where transmission was ongoing.

**Figure 1:**
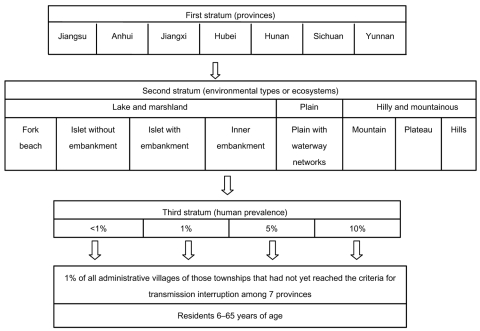
Design of the cluster sampling survey for schistosomiasis, People’s Republic of China.

A stratified cluster random sampling technique with 3 strata was used for village selection. The 7 schistosome-endemic provinces represented the first stratum. Within each province, the second stratum was categorized by characteristics of environmental ecosystems defined and widely used by Chinese health workers, which includes 8 subtypes: 1) fork beach, 2) islet without embankment, 3) islet with embankment, 4) inner embankment in the lake and marshland region, 5) plateau, 6) mountain, 7) hill in the hilly and mountainous region, and 8) waterway networks in the plain region (*20*). Within each ecosystem, estimated local prevalence of *S*. *japonicum*, which was based on results of recent parasitologic examinations, served as the third stratum and used cut-off prevalences of 1%, 5%, and 10%. In each disease-endemic province, ≈1% of administrative villages of the same prevalence class and ecosystem type were randomly selected. One administrative village was randomly selected if the total number of villages was <100 (Figure 1).

All residents 6–65 years of age in selected villages were invited to participate in the study. If the total number of eligible persons was <1,000, residents from neighboring villages with similar ecoepidemiologic characteristics were recruited until >1,000 persons were included.

### Diagnostic Approach

All participants were screened for *S*. *japonicum*–specific immunoglobulin G by using a standardized ELISA (Shenzhen Kangbaide Biotech Co., Shenzhen, People’s Republic of China). Seropositive persons were tested by stool examination. One stool specimen was obtained from each participant, and 3 Kato-Katz thick smear slides were prepared and examined under a light microscope by experienced laboratory technicians (*21*). The number of *S*. *japonicum* eggs was counted on each slide, and the arithmetic mean value was calculated for each person.

In a subsample of villages (n = 25), study participants were interviewed regarding previous infection with *S*. *japonicum* and previous treatment history. Common symptoms, e.g., abdominal pain and diarrhea, were investigated by using a recall period of 2 weeks. Liver and spleen enlargement and the degree of hepatic fibrosis were assessed with a portable ultrasound device. Assessment of illness was performed on supine persons during baseline respiration who had fasted. The fibrotic degree of liver parenchyma was graded between 0 and 3 according to criteria of the World Health Organization–sponsored Cairo Working Group (*22*,*23*).

In 11 villages selected at random from different ecosystems and for different infection levels, seropositive study participants provided 1 stool specimen and 3 Kato-Katz thick smear slides were prepared. Miracidium hatching test after concentration of eggs with a nylon tissue bag was used. Persons with positive results in the miracidium hatching test or with eggs on the slides were identified as having *S*. *japonicum* infections and served as the standard for the diagnosis (*24*–*26*). Comparisons of results of 2 diagnostic approaches enabled calculation of sensitivity of the Kato-Katz technique.

### Data Management and Statistical Analysis

Samples of every new batch of ELISA kits used in the survey were randomly selected and tested with standard serum samples to determine their sensitivity and cross-reactivity with antibodies against hepatitis B virus and *Paragonimus* spp. Rates of false-negative results of serologic tests (compared with the Kato-Katz method) and Kato-Katz thick smears (compared with the hatching test) were calculated.

All data were checked for internal consistency before double entry into standardized databases at provincial institutes. Senior scientists from IPD validated data and established a masterfile from which coverage, compliance, prevalence, and intensity of *S*. *japonicum* infection and total estimates of persons infected were calculated. Statistical analyses were performed by using SAS version 8.0 (SAS Institute Inc., Cary, NC, USA). The following formulas were used to calculate the corrected human infection rate in the village: infection rate *p* = (*x*/*X*)/(1 – *Q*) × (*f*/*F*)/(1 – *R*) and variance *S*^2^*_p_* = *p*(1 – *p*)/(*X* – 1) (1 – *X*/*n*), where *p* is the corrected human infection rate in a sampled village, *S^2^_p_* is the variance of *p*, *x* is the number of the seropositive persons, *X* is the number of eligible persons screened by the serologic test, *f* is the number of stool-positive persons by the Kato-Katz technique, *F* is the number of persons tested by the Kato-Katz technique, *Q* is the false-negative rate of the serologic test, *R* is the false-negative rate of Kato-Katz thick smears, and *n* is the total population in a sampled village.

The χ^2^ test was used to compare *S*. *japonicum* infection rates between provinces, ecosystems, occupational groups, sex and age. Ordinal logistic regression analysis was used to investigate whether there was an association between serologic results and the degree of liver fibrosis, stratified by ecosystem.

### Quality Control

A rigorous quality control system was implemented throughout the study. Blood samples and Kato-Katz thick smear slides from each survey were kept, and 5% of the samples were randomly selected for re-evaluation in the respective provincial institutes. Surveys were repeated in villages where quality control showed a sensitivity <90%. All original data were entered twice, and 10% of original data were compared with submitted databases at IPD. Data from sites where level of agreement was <95% were re-entered.

## Results

### Disease-Endemic Villages, Sampling Scheme, and Compliance

Overall, 17,542 administrative villages with an estimated population of 29,059,194 were classified as endemic for *S. japonicum* in 2004. Appendix Table 1 summarizes the number of villages and persons stratified by different levels of endemicity. Prevalence of *S*. *japonicum* infection was <1% in >50% of villages (n = 9,243) inhabited by >15 million persons. A total of 1,334 villages (7.6%) were classified as settings where infection prevalence was >10%. Estimated population size in these settings was 2,059,211 (7.1%).

The present survey was conducted in 239 villages and included 291,167 persons 6–65 years of age. This corresponds to 1.4% of all schistosome-endemic villages and 1.0% of the population living therein. Compliance to undergo a serodiagnostic test and, among *S. japonucum*–positive persons, to submit a stool sample was high (86.2%).

### Infection Rate and Estimated Number of Persons Infected

Overall, 30,680 (12.2%) of 250,987 participants were positive for *S. japonucum* by ELISA. Of these persons, 94.2% submitted a stool specimen and 9.1% of these specimens were egg positive. Estimated corrected *S*. *japonicum* prevalence, when sensitivity of the Kato-Katz method in *S*. *japonicum*–endemic villages was taken into account, was 2.5%. Prevalence varied considerably by province, from 0.3% (Jiangsu) to 4.2% (Hunan). Prevalence rates above average were found in Hubei (3.8%) and Jianxi (3.1%) Provinces, and prevalence rates in Anhui, Yunnan, and Sichuan Provinces were 2.2%, 1.7% and 0.9%, respectively.

Stratification by ecosystem showed that the highest corrected prevalence was found in the lake and marshland region (3.8%). Overall prevalence in the hilly and mountainous region was 1.1%. Human infection rates among different subtypes of disease-endemic areas showed that the highest rate (7.1%) was seen in the subtype mountain regions, followed by subtypes islets with embankment (4.3%), inner embankment (4.3%), fork beach (3.4%), and islet without embankment (2.7%). Lower prevalence rates were observed in subtypes hill (1.01%) and plateau (0.3%) in the hilly and mountainous regions. A lower prevalence rate of 0.06% was found in the plain region characterized by waterway networks.

**Figure 2:**
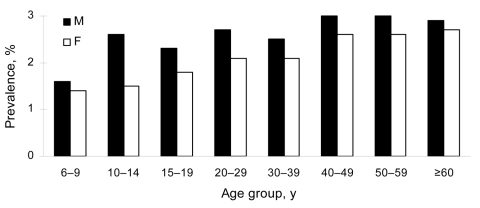
Corrected *Schistosoma japonicum* infection prevalence rates in humans stratified by age and sex, 2004, People’s Republic of China.

As shown in Figure 2, prevalence of *S*. *japonicum* infection in male study participants (2.6%) was higher than that in female participants (2.2%). There was a tendency for prevalence to increase with age, with the highest prevalence found in men 40–50 years of age. Prevalence of infection also varied with occupation. The prevalence in fishermen and boatmen was 3.3%, which was significantly higher than in other professional groups (p<0.001). However, no significant association was found between infection prevalence and educational level (Table).

**Table:** ELISA and Kato-Katz thick smear results for *Schistosoma japonicum* infection prevalence and intensity, stratified by occupation and education, People’s Republic of China*

Characteristic	ELISA		Kato-Katz		Corrected prevalence, %	Geometric mean EPG (infected)	Geometric mean EPG (population)
	No. examined	Positive rate, %	No. examined	Positive rate, %			
Preschool children	848	8.7	65	18.5	0.4	31	0.9
Students	61,430	6.7	3,861	9.7	1.8	37	0.4
Farmers	184,325	14.0	24,285	8.9	2.6	32	0.4
Fishermen and boatmen	1,150	25.0	284	16.5	3.3	54	0.9
Others	3,234	14.0	413	6.5	2.5	30	0.3
Education level							
Illiterate	14,078	18.9	2,559	9.1	2.5	33	0.4
Primary school	119,016	12.2	13,655	9.2	2.5	35	0.4
Junior high school	101,283	11.4	10,917	8.5	2.6	31	0.3
Senior high school	11,616	10.4	1,160	9.3	2.6	31	0.4
Additional education	1,993	7.1	136	10.3	2.3	27	0.4
Unknown	3,001	20.5	481	18.3	2.3	25	0.8
Total	250,987	12.2	28,908	9.1	2.5	33	0.4

*EPG, eggs per gram of feces.

On the basis of survey results, total number of persons infected with *S*. *japonicum* in China in 2004 was estimated to be 726,112 (95% confidence interval [CI] 714,497–737,728). More than 82% of infected persons lived in lake and marshland regions (596,599, 95% CI 585,910–607,287), and most of the remaining persons resided in hilly and mountainous regions (128,720, 95% CI 124,206–133,233).

### Infection Intensity

The geometric mean of infection intensity was 33 eggs per gram (EPG) of feces among egg-positive persons and 0.4 EPG in the general population. Stratified by province, the highest geometric mean among egg-positive persons was found in Jiangxi (56 EPG) and Hunan (0.70 EPG) when the general population was considered. With regard to ecosystem stratification, the highest infection intensity in egg-positive persons was in the plain region with waterway networks (128 EPG), and the highest infection intensity in the entire population was in the lake and marshland regions (0.5 EPG).

Male study participants had a higher infection intensity than females, both among egg-positive persons (34 vs. 30 EPG) and the general population (0.4 vs. 0.3 EPG). No clear trend emerged when stratification was conducted by age, but intensity of infection was generally less variable between age groups in male participants and decreased somewhat with age in female participants. With regard to occupation, fisherman, boatmen, and preschool children had the highest geometric mean infection intensity (0.9 EPG), followed by farmers and students (0.4 EPG). The infection intensity generally decreased with higher educational level (Table).

### Illness and Self-reported Symptoms

Results of *S*. *japonicum*-related illness, as assessed by ultrasonography, and self-reported signs and symptoms, are shown in  Appendix Table 2. Seropositive persons were more likely to report diarrhea over the past 2 weeks than seronegative persons. Degree of liver fibrosis was positively associated with a positive serologic result and with residency in the lake and marshland region. Persons living in the plain region with waterway networks had lower levels of liver fibrosis. No association was found between serologic status and hepatomegaly or splenomegaly.

## Discussion

Results of the third nationwide cluster sampling survey on the epidemiologic status of schistosomiasis provide a comprehensive update on the current extent and distribution of human *S. japonicum* infections in China. The epidemiology of schistosomiasis japonica in China is closely related to local environmental conditions (*20*). Thus, schistosome-endemic areas in China have traditionally been classified according to local environment and local prevalence of infection. The Ministry of Health created different thresholds to classify areas into distinct stages of control. For example, once infection prevalence of *S. japonicum* decreases to <5%, this disease-endemic setting is considered to have reached infection control. Transmission control is declared once local prevalence decreases to <1%. Transmission interruption has been achieved if <2 of 1,000 stool samples examined are egg positive and no new cases have occurred for 5 years. Once this level has been achieved, local elimination can be declared after another surveillance period for 5 years without new infection (*8*,*12*,*27*,*28*).

The 2004 nationwide schistosomiasis survey, which included a correction factor because of the low sensitivity of the Kato-Katz technique, indicated an estimated 726,112 *S*. *japonicum* infections in humans with a small 95% CI. This estimate is lower than other recent estimates of other research groups (*29*–*32*). During the survey, the Kato-Katz technique and the miracidium hatching test were used for fecal examination. It is well known in China that the hatching test is more sensitive than the Kato-Katz technique because the volume of fecal material used for parasitologic detection is several hundred times higher than that used on Kato-Katz slides. However, because immature eggs of schistosomes cannot hatch and egg excretion of *S*. *japonicum* is not uniform, samples that showed no hatching may show eggs in Kato-Katz slides. The hatching test and Kato-Katz technique are mutually complementary and provide higher sensitivity in parasitologic diagnosis of *S*. *japonicum* infection. A combination of these 2 techniques was used in 11 villages as the standard for diagnosis in calculating the false-negative rate of the Kato-Katz technique and is believed to be the most sensitive method for individual and community diagnosis in determining true prevalence (*33*).

The WBLP for schistosomiasis control was started in 1992. It emphasized praziquantel-based control of illness, and progress in disease control was made (*12*). However, in subsequent years, data suggested that schistosomiasis has reemerged (*7**,**9*). Several factors have been suggested as underlying causes, such as unusually severe floods in 1998 (*34*), major ecologic transformations caused by water resource development (*35*), potential effects of climate change (*36**,**37*), market and health sector reforms (*38*), and termination of the WBLP for schistosomiasis control and insufficient attention to control efforts until 2001 (*29*).

Because of conceptual and technical differences, direct comparison is not possible between the current survey and the previous ones conducted in 1989 and 1995. In addition, the current survey covered a wider geographic area because it included all schistosome-endemic areas in which transmission has not yet been interrupted. In previous surveys, the focus was on comparatively smaller areas that had not reached transmission control status, i.e., had a prevalence >1%.

Notwithstanding these differences, several conclusions can be drawn, which in turn are relevant for future design, implementation, and monitoring of schistosomiasis control program in China. The main differences between the second and third nationwide schistosomiasis surveys are summarized in Appendix Table 3. First, estimated number of human cases decreased to 726,112 from 865,084 in the mid-1990s. Consideration of areas where transmission was not controlled showed that the number of infected persons decreased from 847,584 in the second survey to 710,790 in the current survey; a decrease of 16.1%. Second, the number of villages in the areas where transmission was still ongoing has been reduced by 40.3% from 13,911 in 1995 to 8,299 in 2004. Third, the number of people at risk in remaining disease-endemic villages was 13,937,235, a decrease from 22,209,662 in the mid-1990s and a reduction of 37.3%. Finally, areas where schistosomiasis transmission control had not been achieved decreased considerably (Appendix Figure 1, Appendix Figure 2). These findings underscore that the national schistosomiasis control program in China has made further progress over the past decade. Conversely, human prevalence rates in the areas where transmission control has not been achieved increased from 4.9% in 1995 to 5.1% in the present survey, an increase of 3.9%.

Estimated corrected *S. japonicum* prevalence in the current survey averaged over all schistosome-endemic areas was 2.5%. Stratification by province and ecosystem showed spatial heterogeneities. Mean prevalence in villages with an infection rate >1% decreased in Hubei, Hunan, and Yunnan Provinces. In Yunnan, the decrease was most pronounced (–58.6%). Increased prevalences were observed in Jiangsu and Sichuan; prevalence increased from 0.03% to 2.9% in Jiangsu. A fairly constant prevalence was observed in Anhui. Highest corrected prevalence was in the lake and marshland region of eastern and central China, and a low prevalence was found in the mountainous region of Yunnan and Sichuan Provinces. Highest prevalence among all ecosystems was in the mountain ecosystem (7.1%) rather than in the lake and marshland region. This finding probably resulted from decreasing control efforts for schistosomiasis after the end of the WBLP. Corrected prevalence in the plain region with waterway networks was nearly zero. When compared with the previous nationwide survey, prevalence increased by 20.6%–59.0% in fork beach and mountain and hill ecosytems, but decreased by 8.9%–39.7% in other ecosystems.

Typical features of chronic *S*. *japonicum* infection include pathologic changes in the spleen and liver (*39*). Illness assessed by ultrasonography showed changes in ≈70% of all seropositive persons in the plain region with waterway networks, inner embankment, islet without embankment, and hilly ecosystems. Lower rates were found in islets with embankment, plateaus, and mountain ecosystems. In addition, 174 cases of advanced schistosomiasis were detected. Most of these advanced cases were found in the lake and marshland region but 22.4% were in the mountainous and hilly region, a slightly higher percentage than the fraction of the total number of estimated cases in this area.

Additional progress has been made in control of schistosomiasis in China over the past decade. However, currently used control strategies and tools will not eliminate schistosomiasis in certain areas if these strategies are used at the same level of intensity. Applied research on schistosomiasis control is needed to develop new approaches to further reduce infection in these hotspots of transmission. Results of the third national schistosomiasis survey provide a comprehensive overview of the current epidemiology of the disease, which is crucial for the design of the next 5-year plan on schistosomiasis control. This overview will help create at the local level better control programs that include current epidemiologic and socioeconomic conditions to increase their efficiency, address remaining challenges, and avoid reemergence of *S*. *japonicum* infection in areas where it had been controlled (*28*,*31*). By consideration of these challenges and potential risks for transmission of schistosomiasis in China, such as ecosystem changes caused by construction of the Three Gorges Dams on the Yangtze River and effects of global warming (*7*,*18*,*19*,*29*), the Chinese central government has given high priority to control of schistosomiasis and new control goals have been made (*7*,*40*). One goal is to achieve transmission interruption in the hilly and mountainous regions and the plain regions with waterway networks by 2015. The same time frame has been set for transmission control in the lake and marshland region. To attain these goals, renewed efforts are needed to further improve available tools and develop additional control strategies; adapt programs to changing demographic, ecologic, and socioeconomic issues; implement new strategies; and achieve schistosomiasis control in China.

## Supplementary Material

Appendix Table 1 Schistosome-endemic administrative villages and populations in all schistosome-endemic provinces and sampling villages, People's Republic of China*

Appendix Table 2 Schistosomiasis-related signs and symptoms and ultrasonographic results for residents of a randomly selected village per prevalence class and endemic area, stratified by ecosystem and serologic test result, People's Republic of China*

Appendix Table 3 Change between second (1995) and third (2004) nationwide schistosomiasis sampling surveys in villages with a Schistosoma japonicum infection prevalence rate >1%, stratified by location and ecosystem, People's Republic of China

Appendix Figure 1 Regional distribution of schistosomiasis prevalence rates (%) in villages sampled in the second national survey, People's Republic of China, 1995.

Appendix Figure 2 Regional distribution of schistosomiasis prevalence rates (%) in villages sampled in the third national survey, People's Republic of China, 2004.
