# Supplementary material for: Epidemiology of Schistosomiasis in the People’s Republic of China, 2004
Source: Emerg Infect Dis. 2007 Oct;13(10):1470–6. doi: 10.3201/eid1310.061423 (PMC2851518; doi:10.3201/eid1310.061423)
Supplement: Appendix Table 1 — Schistosome-endemic administrative villages and populations in all schistosome-endemic provinces and sampling villages, People's Republic of China* [file 06-1423_appT1-s1.pdf]

Appendix Table 1. Schistosome-endemic administrative villages and populations in all schistosome-endemic provinces and sampling villages, People’s Republic of China*

Province	Infection rate <1%		Infection rate 1%–<5%		Infection rate 5%–10%		Infection rate ≥10%		Total		Sampled villages, no. (%)	Target pop. (age 6–65 y)
	Endemic villages	Pop.	Endemic villages	Pop.	Endemic villages	Pop.	Endemic villages	Pop.	Endemic villages	Pop.		
Anhui	809	1,473,416	732	1,318,480	246	503,901	0	0	1,787	3,295,797	18 (1)	22,555
Jiangsu	868	2,000,710	172	318,060	0	0	0	0	1,040	2,318,770	13 (1.3)	16,435
Jiangxi	282	544,552	699	1,186,190	446	815,582	95	150,901	1,522	2,697,225	23 (1.5)	29,765
Hubei	1,689	2,703,166	1,475	2,645,979	1,043	1,725,977	743	1,133,733	4,950	8,208,855	58 (1.2)	59,533
Hunan	679	1,054,248	1,059	1,574,538	704	1,006,393	390	497,958	2,832	4,133,137	47 (1.7)	51,662
Sichuan	4,831	7,098,038	198	266,698	70	95,346	42	53,924	5,141	7,514,006	68 (1.3)	88,993
Yunnan	85	247,829	72	222,501	49	198,379	64	222,695	270	8,914,04	12 (4.4)	22,224
Total	9,243	15,121,959	4,407	7,532,446	2,558	4,345,578	1,334	2,059,211	17,542	29,059,194	239 (1.4)	291,167

*Pop., population.
