# Supplementary material for: Epidemiology of Schistosomiasis in the People’s Republic of China, 2004
Source: Emerg Infect Dis. 2007 Oct;13(10):1470–6. doi: 10.3201/eid1310.061423 (PMC2851518; doi:10.3201/eid1310.061423)
Supplement: Appendix Table 2 — Schistosomiasis-related signs and symptoms and ultrasonographic results for residents of a randomly selected village per prevalence class and endemic area, stratified by ecosystem and serologic test result, People's Republic of China* [file 06-1423_appT2-s2.pdf]

Appendix Table 2. Schistosomiasis-related signs and symptoms and ultrasonographic results for residents of a randomly selected village per prevalence class and endemic area, stratified by ecosystem and serologic test result, People's Republic of China*

	ELISA		Hepatomegaly (below right midclavicular line, cm)				Hepatomegaly (below xiphoid, cm)			Splenomegaly (below left mid-clavicular line, cm)			Grade of liver fibrosis				
Ecosystem	Result	No.	<2	≥2	≥4	ST	≥3	≥6	ST	<5	≥5	ST	0	I	II	III	ST
Lake and marshland	–	10,014	252	365	51	668	3,079	816	3,895	215	36	251	7,335	2,058	220	64	9,677
	+	1,834	80	128	12	220	715	264	979	52	6	58	1,014	581	154	65	1,814
Plain with waterway networks	–	945	13	45	3	61	416	10	426	9	1	10	802	116	21	6	945
	+	74	0	4	0	4	43	2	45	1	0	1	53	15	6	0	74
Hills and mountains	–	10,061	389	242	39	670	3,222	125	3,347	56	6	62	7,829	1,827	337	42	10,035
	+	2,585	14	29	11	54	352	27	379	8	3	11	1,525	673	326	58	2,582
Total	–	21,020	654	652	93	1,399	6,717	951	7,668	280	43	323	15,966	4,001	578	112	20,657
	+	4,493	94	161	23	278	1,110	293	1,403	61	9	70	2,592	1,269	486	123	4,470

*ST, subtotal; –, negative; +, positive.
