# Supplementary material for: Epidemiology of Schistosomiasis in the People’s Republic of China, 2004
Source: Emerg Infect Dis. 2007 Oct;13(10):1470–6. doi: 10.3201/eid1310.061423 (PMC2851518; doi:10.3201/eid1310.061423)
Supplement: Appendix Table 3 — Change between second (1995) and third (2004) nationwide schistosomiasis sampling surveys in villages with a Schistosoma japonicum infection prevalence rate >1%, stratified by location and ecosystem, People's Republic of China [file 06-1423_appT3-s3.pdf]

Appendix Table 3. Change between second (1995) and third (2004) nationwide schistosomiasis sampling surveys in villages with a *Schistosoma japonicum* infection prevalence rate ≥1%, stratified by location and ecosystem, People's Republic of China

Location	No. villages with prevalence ≥1%			Population			Estimated prevalence, %			
	Second survey	Third survey	Change, %	Second survey	Third survey	Change, %	Second survey	Third survey	Change, %	p value
Province										
Anhui	1,114	978	–12.2	1,890,653	1,822,381	–3.6	4.5	4.2	–7.7	0.178
Jiangsu	1,363	172	–87.4	2,412,173	318,060	–86.8	0.03	2.9	9,665.3	<0.0001
Jiangxi	1,432	1,240	–13.4	2,403,816	2,152,673	–10.5	3.8	5	31.5	<0.0001
Hubei	4,171	3,261	–21.8	7,096,742	5,505,689	–22.4	7.2	5.6	–22.1	<0.0001
Hunan	2,582	2,153	–16.6	3,389,332	3,078,889	–9.2	7.8	5.7	–26.9	<0.0001
Sichuan	2,977	310	–89.6	4,166,074	415,968	–90	1.9	4	107.6	<0.0001
Yunnan	272	185	–32	850,872	643,575	–24.4	6	2.5	–58.6	<0.0001
Ecosystem										
Lake and marshland										
Fork beach	402	383	–4.7	630,047	630,537	0.08	7.7	12.3	59.0	<0.0001
Islet without embankment	1,851	1,262	–31.8	3,751,536	2,391,860	–36.24	6	3.6	–39.7	<0.0001
Inner embankment	2,056	1,699	–17.4	2,772,536	2,585,846	–6.73	7.1	5.2	–27.7	<0.0001
Subtotal	8,606	6,672	–22.5	14,113,494	10,992,519	–22.11	6.6	5.7	–14.4	<0.0001
Plain with waterway networks	912	63	–93.1	1,355,261	112,744	–91.68	0.02	–	–	–
Hills and mountains										
Plateau	1,413	196	–86.1	2,580,274	597,340	–76.85	2.1	1.6	–20.0	0.0019
Mountain	725	106	–85.4	947,054	207,579	–78.08	5.4	6.6	20.6	0.0001
Hill	2,255	1,262	–44	3,213,579	2,027,053	–36.92	2.1	3	40.9	<0.0001
Subtotal	4,393	1,564	–64.4	6,740,907	2,831,972	–57.99	2.8	3	7.4	0.0181
Total	13,911	8,299	–40.3	22,209,662	13,937,235	–37.3	4.9	5.1	4.3	0.0047
