# Supplementary figures and images for: Epidemiology of Schistosomiasis in the People’s Republic of China, 2004
Source: Emerg Infect Dis. 2007 Oct;13(10):1470–6. doi: 10.3201/eid1310.061423 (PMC2851518; doi:10.3201/eid1310.061423)

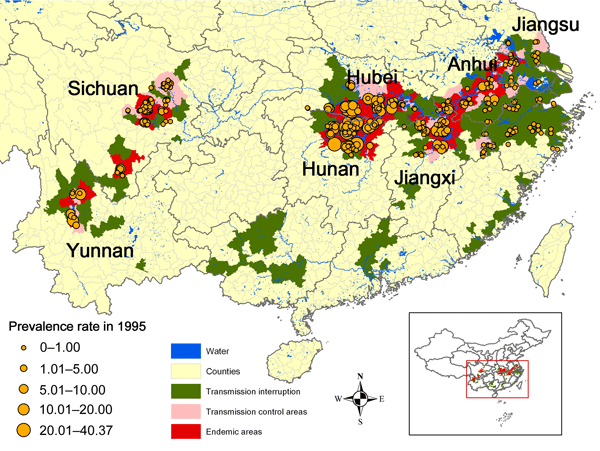

Supplement: Appendix Figure 1 — Regional distribution of schistosomiasis prevalence rates (%) in villages sampled in the second national survey, People's Republic of China, 1995. [file 06-1423_appF1-s4.gif]
